# Thermal and Medium Stability Study of Polyvidone-Modified Graphene Oxide-Coated Gold Nanorods with High Photothermal Efficiency

**DOI:** 10.3390/nano12193382

**Published:** 2022-09-27

**Authors:** Thabang Calvin Lebepe, Oluwatobi Samuel Oluwafemi

**Affiliations:** 1 Department of Chemical Science, University of Johannesburg, Johannesburg 2028, South Africa; 2 Centre for Nanomaterials Sciences Research, University of Johannesburg, Johannesburg 2028, South Africa

**Keywords:** gold nanorods, graphene oxide, surface modification, colloidal stability, photothermal

## Abstract

Coating gold nanorods (AuNRs) with different materials, such as polymers and graphene-based materials, has improved their biocompatibility. However, these materials have been shown to cause the instability of AuNRs in thermal and culture mediums. In addressing this issue, we herein report the synthesis, thermal and culture medium stability, and photothermal profiling of Polyvidone (PVP)-modified graphene oxide (GO)-coated AuNRs (mGO@AuNRs). The AuNRs, with a size of 40.70 nm × 9.16 nm and absorbing at 820 nm, were coated with PVP, GO, and mGO. The colloidal stability of the nanocomposites was tested in three commonly used cell culture mediums: the Roswell Park Memorial Institute 1640 (RPMI-1640), Dulbecco’s Modified Eagle Medium, (DMEM) and Dulbecco’s phosphate-buffered saline (PBS) using UV-Vis-NIR and dynamic light scattering. The GO-based nanocomposites were stable compared to PVP@AuNRs and AuNRs in all mediums. The photothermal profiling of mGO@AuNRs showed higher heat production, with the photothermal conversion efficiency of 54.8%, which is higher than the bare AuNRs, GO@AuNRs, and PVP@AuNRs. In addition, the mGO@AuNRs also showed good thermal stability at 70 °C for more than 24 h. These results present the dual coating of PVP and GO as excellent stabilising agents for AuNRs with good photothermal profiling.

## 1. Introduction

The use of nanostructure and their nanocomposite has grown widely over the years in different applications, such as theranostics [1,2,3,4], catalysts [5], sensors [2] and others, depending on their properties. Nanostructures come in different ranges of shapes and sizes. The gold nanostructures, in particular, have been widely researched, especially the gold nanorods (AuNRs). The applications of AuNRs are increasing day by day, with applications ranging from biosensing, photothermal therapy, bioassays, and biomedical imaging to drug delivery, due to their exceptional optical properties. Their anisotropic shape offers distinct plasmonic modes in the near-infrared (NIR) range which can be tuned from 650–1350 nm, making them an ideal material for phototherapeutic treatments [2,6]. These optical properties make AuNRs a good photothermal agent, as they can be used to treat deeply embedded tumours. Nonetheless, AuNRs are synthesised using cetyltrimethylammonium bromide (CTAB), which induces toxicity in biological applications.

Currently, different coating materials, such as polymers (polyvinylpyrrolidone (PVP)) [6,7,8,9,10,11], biomolecules [12,13,14], and graphene-based materials (graphene oxide (GO)) [10,15,16,17,18,19], have been employed to reduce the toxicity of AuNRs. Among these materials, graphene-based AuNRs has been shown to be more effective as they minimise toxicity and simultaneously increase photothermal efficiency, in particular, GO [10,15,16,17,18,20].

GO is an exfoliated graphene sheet with carbon-bonded oxygen functional groups, such as epoxy, hydroxyl, carbonyl, and carboxylic [2,19,20,21,22]. It is biocompatible and easy to incorporate with different nanomaterials because of its functional groups [19,23]. However, combining various nanomaterials straight to the GO has resulted in nanoparticle aggregation [2]. It has been reported that functionalising GO with polymers, such as PVP [24,25,26,27,28,29], poly-L-lysine (PLL) [1], and polyvinyl alcohol (PVA) [30], can increase the functional groups on its surface and reduce the aggregation of the incorporated nanoparticle, improve its solubility, and increase it biocompatible.

Zhang et al. [31] reported the fabrication of core–shell-structured amorphous zinc oxide on gold NPs integrated onto the planar structure of pegylated graphene oxide (GO@Au-His@a-ZnO NCs), with an enhanced photothermal efficiency (PCE = 38.0%), compared to AuNRs. In another study, the photothermal effect of the AuNRs coated with polyethylene glycol (PEG)-modified GO nanocomposites showed a high heat production when exposed to a laser with a 60 W/cm^2^ for 5 min, significantly enough to kill ≈40% of cancer cells [7]. Sun and co-workers [16] reported that the AuNRs coated with GO-modified polystyrene sulfonate (PSS) can heat up to 49.9 °C after irradiation with an 808 nm laser for 6 min without hindering its optical and morphological [16]. In another development, AuNRs coated with natural polymer gum arabic-functionalised GO has also been reported to increase AuNRs photothermal profiling. In a recent study, Zhang et al. [32] synthesised L-cysteine-reduced graphene oxide-coated AuNRs (AuNR@Lcyst-rGO) to improve the photothermal effect and reduce the cytotoxicity [32]. In another study, Qi et al. [10] reported the immobilisation of AuNRs onto the surface of PEGylated graphene oxide (GO-PEG) via polydopamine (PDA), as an excellent photostable nanocomposite with PCE of 36.10% for efficient drug delivery [10]. The PVP-modified GO has also been used to improve the AuNRs cytotoxicity and increase its photothermal profiling [33,34]. Though these modifications produced materials with improved PCE and reduced cytotoxicity, compared to the bare AuNRs, information about their thermal, as well as their medium stabilities, is still unavailable. This is essential for their biological applications and need to be investigated.

Herein, we reported the interaction of the dual-coated AuNRs (mGO@AuNRs) with different biological culture mediums; Roswell Park Memorial Institute (RPMI-1640), phosphate-buffered saline (PBS) and Dulbecco’s Modified Eagle Medium (DMEM). This will assist in understanding the nanomaterial’s physicochemical properties when interacting with the complex biological media, comprising electrolytes, proteins, lipids, and many more [35]. The RPMI-1640 and DMEM are the commonly used medium for mammalian cell culture. The difference between the two mediums is that the RPMI-1640 contains the reducing agent glutathione and high concentrations of vitamins, such as biotin, vitamin B_12_, and para-aminobenzoic acid. The Dulbecco’s phosphate-buffered saline (PBS) is a buffer solution used for different cell culture applications, such as washing cells before dissociation, transporting cells or tissue samples, diluting cells for counting, and preparing reagents/drugs for inoculation [11]. The photothermal profiling of the as-synthesised dual-coated AuNRs (mGO@AuNRs) was also investigated and compared with the bare AuNRs, GO-coated AuNRs, and PVP@AuNRs. These results present the dual coating of PVP and GO as excellent stabilising agents for AuNRs with good photothermal profiling for different photothermal applications.

## 2. Materials and Methods

### 2.1. Materials

Hydrogen tetra-chloroauric hydrate (HAuCl_4_ xH_2_O, 99.9%), sodium borohydride (NaBH_4_, 99%), silver nitrate (AgNO_3_, 99%), cetyltrimethylammonium bromide (CTAB, ≥99%), Hydroquinone (HQ, 99%), sodium oleate, (NaOL, ≥99%), hydrochloric acid (HCl, (12.1 M)), Dulbecco’s Modified Eagle Medium (DMEM), Dulbecco’s phosphate-buffered saline (PBS), Roswell Park Memorial Institute (RPMI) medium, graphite powder, sulfuric acid (H_2_SO_4_, 95.0–98.0%), sodium nitrate (NaNO_3_, 99%), potassium permanganate (KMnO_4_, 99%), peroxide (H_2_O_2_, 30%), and sodium hydroxide (NaOH, ≥98%) were purchased from Sigma-Aldrich, (Kempton Park, South Africa). All glassware used in the experiments was cleaned, washed thoroughly with MilliQ water (15.0 MΩ cm @ 25 °C), and dried before use.

### 2.2. Methods

#### 2.2.1. Synthesis of Graphene Oxide

GO was prepared using a modified Hummers’ method [36]. Briefly, a concentrated ice-cold H_2_SO_4_ was added to a combination of graphite flakes and sodium nitrate under continuous stirring in an ice bath. This was followed by the slow addition of KMnO_4_ to the solution. The solution was then warmed to 35 °C while stirring for 30 min. The distilled water was added slowly afterwards, and the solution temperature was increased to 95 °C. After 30 min, the solution turned deep brown colour. The solution was allowed to cool to room temperature before adding H_2_O_2_ to end the reaction. The solution was allowed to settle, and the settled product was washed with distilled water several times to neutralise the pH. The obtained graphite oxide powder was exfoliated using ultrasonication to get homogeneous GO dispersion.

#### 2.2.2. Synthesis of Gold Nanorods

The AuNRs were synthesised using our previous method [37]. Briefly, 6.0 mL of freshly prepared ice-cold 0.010 M NaBH_4_ (prepared in NaOH (0.01 M)) was added to a solution containing 2.5 mL of HAuCl_4_·xH_2_O (0.01 M) and 95.0 mL of CTAB (0.10 M). The solution was stirred for 10 min and kept unstirred for an hour at room temperature. The growth solution was prepared by mixing 100 mL of HAuCl_4_·xH_2_O (0.01 M), 400 mL of CTAB (37.0 mM) and NaOL (0.23 M) mixture, and 2 mL of freshly prepared AgNO_3_ (0.10 M), respectively, under gentle stirring for 15 min. This was followed by adding 1.80 mL HCl (1.0 M) and 25.0 mL hydroquinone (0.10 M) at an interval of 15 min. Lastly, 100 mL of the prepared seed solution was added and stirred for 2 min. The solution was left unstirred for 20 h at room temperature. After 20 h, the AuNRs were collected by centrifugation at different speeds to remove excess CTAB and Au nanoparticles.

#### 2.2.3. Synthesis of GO@AuNRs

The GO@AuNRs nanocomposite was prepared by adding 100 µL GO to 2 mL AuNRs and stirred for 24 h. The nanocomposite was then centrifuged to remove excess GO and AuNRs at 4500 rpm. The sediment was collected and resuspended.

#### 2.2.4. Synthesis of mGO@AuNRs

The synthesis of mGO@AuNRs nanocomposite was done by adding 16 mg of PVP to GO dispersion (4 mL, 0.1 mg/mL) under continuous stirring for 30 min. After 30 min, AuNRs (2 mL, OD 2) was introduced to the modified GO solution with stirring for 24 h at ambient temperature. The product was centrifuged three times to eradicate excess PVP and AuNRs. The final sediment was dissolved in 2 mL of distilled water.

#### 2.2.5. Synthesis of PVP@AuNRs

The PVP@AuNRs was prepared by adding 16 mg PVP in 4 mL distilled water and stirred for 30 min to dissolve the PVP before adding 2 mL of AuNRs. The solution was stirred for 24 h at room temperature. The resulting solution was centrifuged three times and redispersed in 2 mL of distilled water.

#### 2.2.6. Medium Stability Study

The medium stability of all the as-synthesised materials was tested by following Mahmoud et al., 2021 [38] method with slight modification. In brief, 1 mL of PBS/RPMI/DMEM medium solution and 1 mL of each sample were added to a vial, and these vials were placed in an incubator set at 37 °C. Aliquots were taken at different time intervals (15, 30, 45, 60, 75, 90 min, and 24 h) and analysed with the UV-Vis-NIR after each incubation.

#### 2.2.7. Photothermal Profiling

The photothermal profiling of AuNRs, PVP@AuNRs, GO@AuNRs, and mGO@AuNRs was measured by placing 1.5 mL solution of each sample into a separate 1.5 mL Eppendorf tube followed by irradiation using 808 nm laser with 1.27 W·cm^−2^ power density. The temperature changes were measured using the RS-1384 PRO thermocouple and the FLIR E4 thermal camera. The photothermal conversion efficiency (PCE) was evaluated by irradiating 1.5 mL of each solution with an 808 nm power density of 1.27 W·cm^−2^. The temperature of the dispersions was recorded every 30 s using a thermocouple. The PCE was calculated using Equation (1) following Li et al.’s calculation with modifications [39].

(1) $$\mathrm{PCE}=\frac{\mathrm{hS}\left(\Delta T_{\max}\right)-Q_{\mathrm{Dis}}}{I\left(1-10^{-A_{808}}\right)}$$

where h (mW (m^−2^ °C^−1^)) is the heat transfer coefficient, S (m^2^) is the surface area of the container, ΔT_max_ (°C) is the difference between equilibrium temperature and the ambient temperature of the surroundings. To get hS, Equation (2) was used, where a time constant τ_s_ in the spontaneous cooling system is introduced, given as

(2) $$\tau_{s}=\frac{\sum_{i}m\cdot \;C}{\mathrm{hS}}$$

where m is the mass (2 g), the specific heat (C) of water is 4.2 J.g^−1^.°C^−1^, and the sample system time constant τs (s) was obtained from the natural logarithm of temperature change versus time data from Equation (3):

(3) $$t=\tau_{s}\ln\left(\theta \right)$$

where t is the cooling time after switching off the laser, and θ is the temperature-related variable.

#### 2.2.8. AuNRs and Modified AuNRs Thermal Stability Method

The thermal stability of all the as-synthesised materials was investigated by following Centi et al. method with slight modification [40]. Shortly, 2 mL of the different solutions were added to a vial, and these vials were placed in an oven set at 70 °C. The samples were heated at different time intervals (15, 30, 45, 60, 75, 90, 120, 150, and 180 min) and analysed with the UV-Vis-NIR after each time interval.

#### 2.2.9. Characterisation Techniques

The UV-Vis-NIR JASCO V-770 spectrophotometer (JASCO Corp., Tokyo, Japan) was used to obtain the absorption spectra. JEOL 2010 high-resolution transmission electron microscopy (HRTEM, JEOL 2010, 200 KV, Tokyo, Japan) was used to determine the morphology of the as-synthesised materials. ImageJ software (Version 1.51, National Institute of Heath, Maryland, MD, USA) was used to measure the particle size distributions and aspect ratios of the AuNRs from TEM images. The hydrodynamic dimensions were obtained from a Microtrac MRB’s NANOTRAC Wave II (Microtrac MRB, Duesseldorf, Germany). The FTIR spectra were obtained from the Perkin Elmer spectrum-II UATR spectrometer (Perkin Elmer, Midrand, South Africa). A dst11-LUMICS-808 nm 27 W continuous Nd: YV04 air-cooled laser system (OsTech e. K., Berlin, Germany) with an optical fibre to deliver an 8 mm beam diameter was used for the irradiation.

## 3. Results

### 3.1. Optical and Structural Characterisations

**Figure 1:**
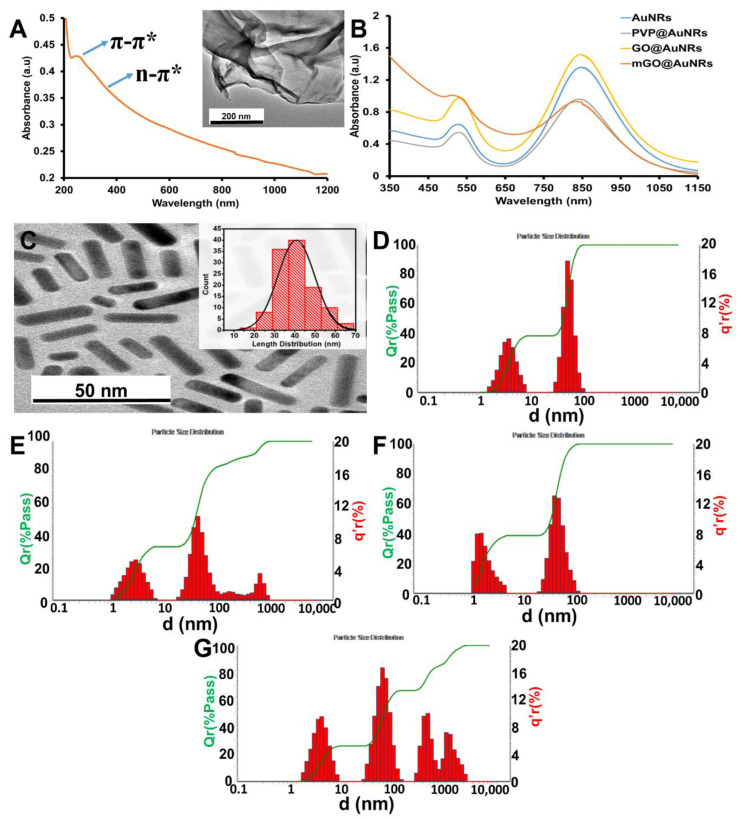
UV-Vis-NIR spectra of (**A**) GO (Inset: GO TEM image scale 200 nm) and (**B**) UV-Vis-NIR spectrum of AuNRs, PVP@AuNRs, GO@AuNRs, and mGO@AuNRs. (**C**) TEM image of AuNRs (inset: Size distribution, TEM image scale 100 nm). Hydrodynamic size distribution of (**D**) AuNRs, (**E**) GO@AuNRs, (**F**) PVP@AuNRs, and (**G**) mGO@AuNRs.

**Scheme 1:**
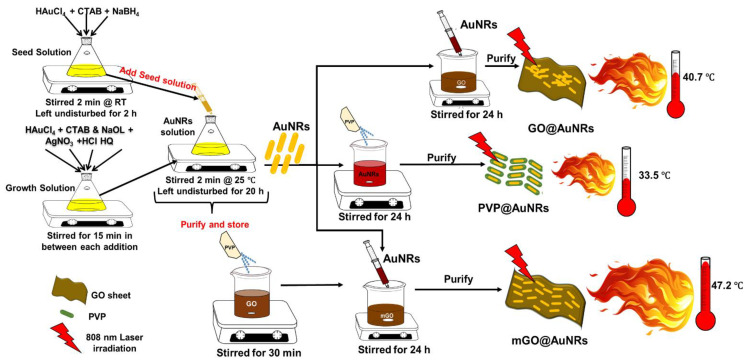
Schematic diagram of the synthesis and photothermal profiling of AuNRs, PVP@AuNRs, GO@AuNRs, and mGO@AuNRs.

GO was prepared using the Hummers method and then modified with PVP to increase the aqueous dispersibility [28,29]. Figure 1A shows the π-π* carbon-carbon and *n*-π* oxygen-carbon transitions of the as-synthesised GO [36]. The AuNRs were synthesised using a binary surfactant seed-mediated method with hydroquinone as the reducing agent [37]. Scheme 1 shows the synthetic diagram for AuNRs, PVP@AuNRs, GO@AuNRs, and mGO@AuNRs. UV-Vis-NIR spectroscopy and TEM were used to confirm the size and optical properties of the as-synthesised AuNRs. Figure 1B shows the UV-Vis-NIR spectrum of the as-synthesised AuNRs, with two peaks representing the transverse surface plasmon resonance (TSPR) and longitudinal surface plasmon resonance (LSPR) at 512 nm and 820 nm, which are associated with AuNRs, respectively. The TEM image showed rod-shaped particles with a size range of 40.70 ± 9.1 nm × 9.16 ± 1.3 nm and an aspect ratio of 4.4 (Figure 1C). The size range was measured using ImageJ software, and the data are shown in Figure 1C, inset. The AuNRs solution also had a small number of spherical shapes with an average size of 15 nm. The TEM micrographs of GO@AuNRs and mGO@AuNRs show the presence of AuNRs on the GO sheet (Figure S1A,B). The results show that, unlike mGO@AuNRs, the images of GO@AuNRs (without modification) showed aggregation of the AuNRs. The DLS results show similar size distribution, with a hydrodynamic size of 44.6 nm (Figure 1D). The hydrodynamic size distribution of GO@AuNRs and mGO@AuNRs had an extra peak at around 1000 nm due to the GO sheet (Figure 1E,G). The hydrodynamic size of PVP@AuNRs did not have any additional peaks. It was similar to the AuNRs (Figure 1F).

The UV-Vis-NIR spectrum of GO@AuNRs composites shows the presence of AuNRs peaks (Figure 1B). This indicates that the incorporation of AuNRs on the GO sheet did not affect their shape. A similar result was observed with PVP@AuNRs. However, the AuNRs coated with modified GO (mGO@AuNRs) showed a slight blue shift, compared to the bare AuNRs.

**Figure 2:**
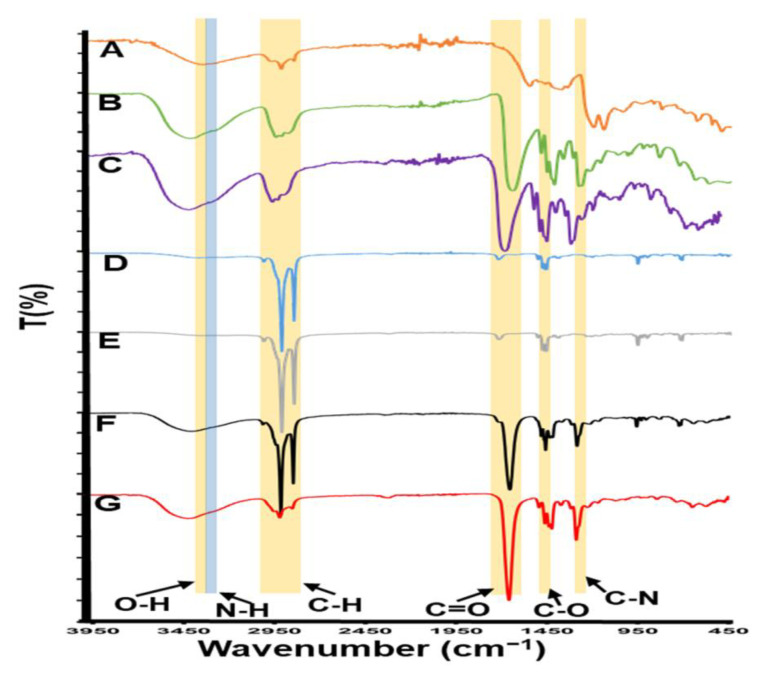
FTIR spectra of (**A**) GO, (**B**) PVP, (**C**) mGO, (**D**) AuNRs, (**E**) GO@AuNRs, (**F**) PVP @AuNRs, and (**G**) mGO@AuNRs.

The surface chemistry of all the as-synthesised materials was investigated using FTIR. The FTIR spectrum of AuNRs shows characteristic peaks of CTAB with long C-H asymmetric and symmetric stretching vibrations at 2916 and 2849 cm^−1^, respectively (Figure 2D). The corresponding C-H bending vibrations with the splitting is at 1473 and 1463 cm^−1^_,_ while the C-H (CH_3_−N^+^) asymmetric and symmetric bending vibrations are at 1487 and 1431 cm^−1^, respectively (Figure 2D) [20]. The GO spectrum shows O-H stretching at 3416 cm^−1^, C=O vibration shoulder peak at 1761 cm^−1^, aromatic C=C stretching at 1654 cm^−1^, and epoxy and carboxyl vibrations between 1230 to 1320 cm^−1^ (Figure 2A) [36,41]. The GO@AuNRs composite showed more of the AuNRs peak than the GO (Figure 2E).

The FTIR spectra of GO, PVP, and mGO showed that PVP chains have completely masked GO sheet surfaces (Figure 2A–C). This could be attributed to the fact that PVP and mGO vibrations are similar, with both spectra showing two stretched peaks at 1670 and 1283 cm^−1^, corresponding to C=O and C–N of PVP, respectively (Figure 2B,C). Figure 2F shows a spectrum of PVP@AuNRs with strong C=O vibrations at ~1667 cm^−1^ and C-O and C-N bands at ~1240 cm^−1^ and 1281 cm^−1^, respectively, attributed to the presence of PVP. Similar observations have been reported by Fan et al. [42]. The FTIR spectrum of mGO@AuNRs (Figure 2G) demonstrated the characteristic peaks of mGO at ~3424 cm^−1^, 2860–2925 cm^−1^, ~1667 cm^−1^, ~1240 cm^−1^, and 1281 cm^−1^, corresponding to the O–H, C–H, C=O, C–O and C-N band vibrations, respectively. In addition, the alkyl C-H stretching vibration bands at ~2850 and ~2918 cm^−1^, corresponding to the CTAB capping agent on the AuNRs were also visible but not stretched, as seen in the AuNRs spectrum.

### 3.2. Media Stability

**Figure 3:**
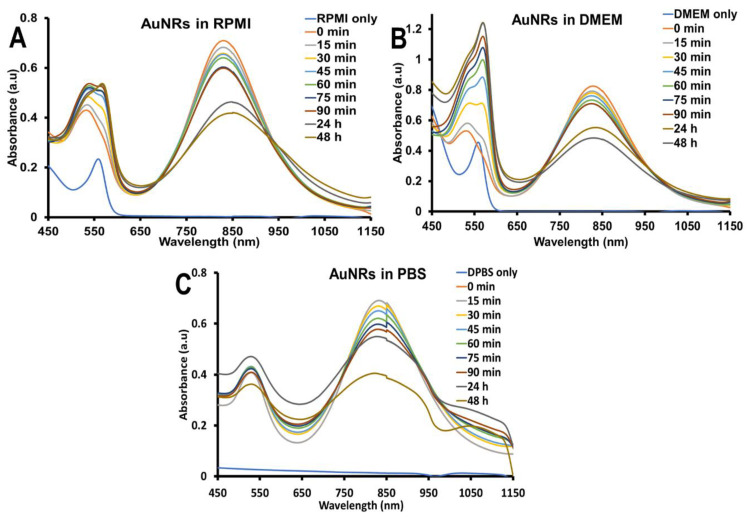
The absorption spectrum of AuNRs in (**A**) RPMI at ratio volume, (**B**) DMEM, and (**C**) DPBS at volume ratio (1:1).

The UV-Vis-NIR was used to monitor the effect of RPMI on the AuNRs’ size and shape. The LSPR absorbance decreased gradually with time with no indication of any blue shifting in the LSPR peak position up to 90 min (Figure 3A). The TSPR peak absorbance increased with time as an indication of the increasing sphere shapes with a shoulder from the phenol-red (560 nm). After 24 h, the LSPR peak position red-shifted, which could be attributed to the interaction between the AuNRs and the protein in the RPMI. Similar results were observed in DMEM + AuNRs (Figure 3B), even though the phenol-red peak was more pronounced after 45 min, when compared to RPMI + AuNRs. The study was also carried out in Dulbecco’s phosphate-buffered saline (PBS) at the same ratio as RPMI and DMEM (1:1). Figure 3C shows the AuNRs LSPR peak with similar reducing absorbance as previous mediums. These results show that the cell culture’s medium affects the AuNRs. Thus, it is essential to find good stabilising agents.

**Figure 4:**
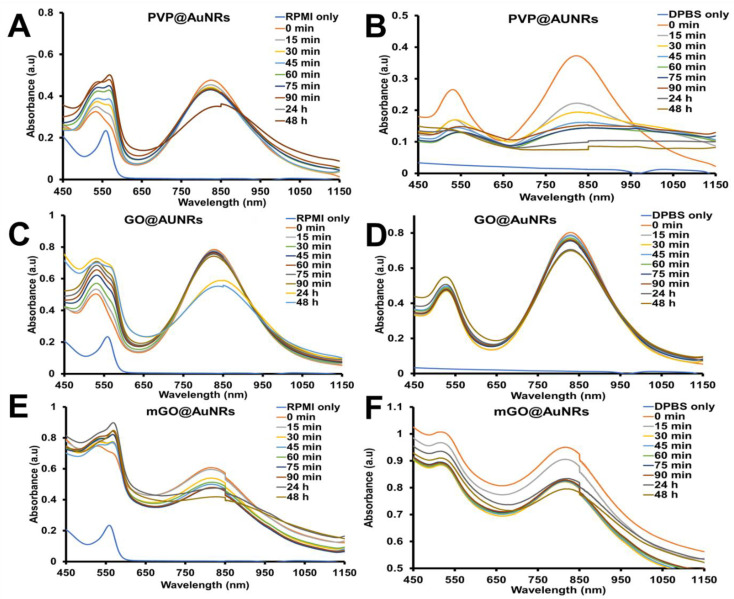
UV-Vis-NIR spectrum of (**A**,**B**) PVP @AuNRs, (**C**,**D**) GO@AuNRs, and (**E**,**F**) mGO@AuNRs, in RPMI and PBS, respectively, at different time intervals.

The coated AuNRs were also tested in RPMI and DPBS at a ratio of 1:1 and at different time intervals. The PVP@AuNRs in RPMI were slightly blue-shifted for the first 90 min and red-shifted after 24 h (Figure 4A). The blue-shifting could be attributed to the binding of PVP with the biomolecules. This exposed the AuNRs to the proteins present in the medium and their subsequent adsorption on the surface of AuNRs due to distinct binding affinities [43]. This adsorption resulted in the red-shifting absorption seen after 24 h. The same behaviour can be observed with the GO@AuNRs and mGO@AuNRs (Figure 4C,E). The samples behave differently in PBS, with PVP@AuNRs the most unstable composite when compared to the others (Figure 4B). The GO@AuNRs was the most stable with a slight reduction in absorbance after 48 h (Figure 4D). Though the mGO@AuNRs was also stable, however, the presence of PVP in the GO was attracting the biomolecules on the surface of the sheet, which affected the stability of AuNRs on the surface of the GO (Figure 4F). However, the presence of PVP is necessary to prevent the aggregation of AuNRs, which usually occurs when AuNRs is attached to bare GO (Figures S1A, S2 and S3). The stability of GO nanocomposite can be attributed to the GO, as it has been described as preventing the protein from absorbing in physiological environments [44].

### 3.3. Photothermal Conversion and Thermal Stability of AuNRs and Modified AuNRs

**Figure 5:**
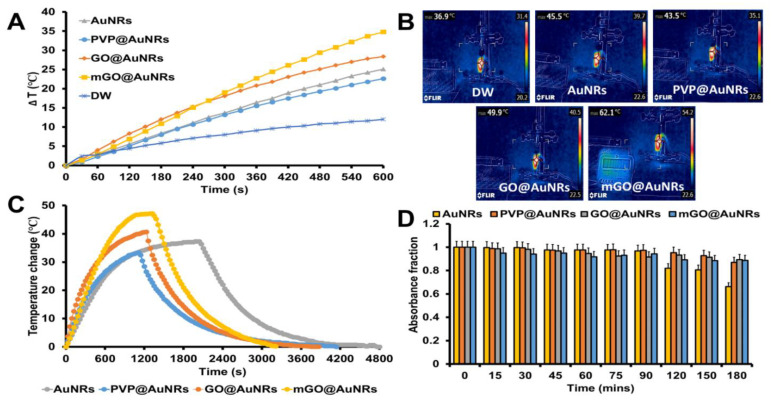
(**A**) Photothermal response of water, AuNRs, PVP@AuNRs, GO@AuNRs, and mGO@AuNRs after irradiation with laser at 1.27 W.cm^−2^ for 10 min. (**B**) Thermal IR images of water, AuNRs, PVP@AuNRs, GO@AuNRs, and mGO@AuNRs after irradiation with laser at 1.27 W.cm^−2^ for 10 min. (**C**) Temperature profile generated by AuNRs, PVP @AuNRs, GO@AuNRs, and mGO@AuNRs after irradiation with 808 nm laser at 1.27 W.cm^−2^ (**D**) LSPR absorbance fraction after 70 °C incubation of AuNRs, PVP@AuNRs, GO@AuNRs, and mGO@AuNRs at different times (0, 15, 30, 60, 75, 90, 120, 150, and 180 min) intervals.

The photothermal profiling studies of PVP@AuNRs, GO@AuNRs, and mGO@AuNRs were compared to the unmodified AuNRs. The photothermal results were recorded using a thermocouple and thermal camera after irradiating for 10 min. The thermocouple in Figure 5A shows that the mGO@AuNRs have the highest temperature change of 34.8 °C, followed by GO@AuNRs (28.4 °C), bare AuNRs (25.1 °C), PVP@AuNRs (22.6 °C), and lastly deionised water (DW, 12 °C). The highest temperatures of mGO@AuNRs and GO@AuNRs were due to the enriched photon absorption caused by GO sheets when irradiated with a laser [15,20,45]. The thermal camera images in Figure 5B show the actual temperature captured at 10 min irradiation for all the samples. The heat produced by all the nanocomposites (from the thermal camera and thermocouple temperature) in 10 min is enough to destroy cancer cells and pathogens [3,46], hence making them an ideal photothermal anticancer and antibacterial agent. The photothermal conversion efficiency of AuNRs, PVP@AuNRs, GO@AuNRs, and mGO@AuNRs were calculated using temperature response maximum during irradiation before switching off the laser (Figure 5C), and the natural logarithm of temperature change versus time data, which was obtained from the cooling cycle (Figure S4) and Equation (1). The data are tabulated in Table 1 with the max temperature before cooling. The following photothermal conversion efficiencies were obtained 39.2%, 21.5%, 37.8%, and 54.8% for AuNRs, PVP@AuNRs, GO@AuNRs, and mGO@AuNRs, respectively. The lower photothermal conversion efficiency of PVP@AuNRs could be attributed due to the weak interaction between PVP molecules and AuNRs, which caused the dissociation of PVP molecules from the surface of AuNRs during the irradiation. The higher percentage of mGO@AuNRs was due to the enhanced photon absorption by a high amount of GO. It is worth noting that a high amount of GO was also used in the GO@AuNRs, similar to mGO@AuNRs. However, the AuNRs were aggregating in the composite. The photothermal conversion efficiency of mGO@AuNRs indicates that it has an excellent photothermal agent, compared with the literature-reported nanocomposite agents (Supplementary Material Table S1).

The thermal stability of PVP@AuNRs, GO@AuNRs, and mGO@AuNRs was investigated at 70 °C at different time intervals (0, 15, 30, 60, 75, 90, 120, 150, and 180 min) by monitoring the LSPR absorbance (Figure 5B). The 70 °C temperature was chosen based on the actual maximum temperature of mGO@AuNRs before switching off the laser in the photothermal conversion experiment. The AuNRs were not stable at 70 °C after 90 min in the incubator, and the LSPR peak was reduced by 35% after 180 min. The PVP@AuNRs was only reduced by 14%, followed by GO@AuNRs by 12%, and the most stable was mGO@AuNRs with only an 11% decrease. The results present the mGO@AuNRs as an ideal material for photothermal cancer therapy, photothermal anti-pathogen therapy, and wound healing because of its high photothermal conversion and thermal stability.

## 4. Conclusions

In conclusion, the synthesis, thermal and culture medium stability, and photothermal profiling of AuNRs dual-coated with polyvidone (PVP) and graphene oxide (GO) have been reported. The GO was successfully synthesised using the Hummers method, while the AuNRs was synthesised using the seed-mediated method. The AuNRs was coated with PVP-, GO-, and PVP-modified GO using simple electrostatic interaction. The coating of AuNRs with different materials (PVP-, GO-, and PVP-modified GO) was confirmed using UV-Vis-NIR, TEM, DLS and FTIR, which showed the characteristics of both the AuNRs and the coating materials. The GO@AuNRs without modification was more stable than the mGO@AuNRs and PVP@AuNRs. This was due to the attraction of proteins to PVP. Coating with PVP only was shown to affect the AuNRs stability in all the mediums. However, it did not show a significant effect when incorporated into the mGO@AuNRs. Furthermore, the mGO@AuNRs had higher heat production upon irradiation than the other modified AuNRs and the bare AuNRs. The photothermal conversion efficiency also confirms the results, with mGO@AuNRs having the highest percentage. In addition, the modified AuNRs were more thermally stable than the bare-AuNRs. These results show that the modified GO can be used to stabilise the nanomaterials and improve their photothermal properties upon irradiation while keeping them stable for bio-photothermal applications, such as photothermal cancer therapy, photothermal anti-pathogen, and wound healing.

## Figures and Tables

**Table 1:** Tabulated summary of temperature change and the photothermal conversion efficiency.

Sample	Max. ΔT (°C)	Linear Fit (Time vs. *ln*(ϴ))	R^2^	η
AuNRs	37.3	y = 564.83x − 132.4	0.9949	39.2%
PVP@AuNRs	33.5	y = 710.32x − 16.637	0.9982	21.5%
GO@AuNRs	40.7	y = 566.64x − 101.44	0.9979	37.8%
mGO@AuNRs	47.2	y = 484x − 225.39	0.9864	54.8%

## Data Availability

The data presented in this study are available on request from the corresponding author.

## Supplementary Materials

The following supporting information can be downloaded at: https://www.mdpi.com/article/10.3390/nano12193382/s1, Figure S1. TEM images of GO@AuNRs and mGO@AuNRs; Figure S2. DLS of AuNRs, PVP @AuNRs, GO@AuNRs and mGO@AuNRs, in RPMI at 0 min and 48 h. Figure S3. DLS of AuNRs, PVP @AuNRs, GO@AuNRs and mGO@AuNRs, in PBS at 0 min and 48 h. Figure S4. The time constant for heat transfer of AuNRs, PVP @AuNRs, GO@AuNRs, and mGO@AuNRs by applying the natural logarithm of temperature change versus time data acquired from the cooling. Table S1. Power density and photothermal conversion efficiency (%) of different nanocomposites irradiated with 808 nm laser. References [10,31,47,48,49] were cited in Supplementary Materials.
