# Supplementary material for: Thermal and Medium Stability Study of Polyvidone-Modified Graphene Oxide-Coated Gold Nanorods with High Photothermal Efficiency
Source: Nanomaterials (Basel). 2022 Sep 27;12(19):3382. doi: 10.3390/nano12193382 (PMC9565574; doi:10.3390/nano12193382)
Supplement: Supplementary file 1 [file nanomaterials-12-03382-s001.zip › nanomaterials-1916325-supplementary.pdf]

nanomaterials

Article

Thermal and Medium Stability Study of Polyvidone-Modified Graphene Oxide-Coated Gold Nanorods with High Photothermal Efficiency

Thabang Calvin Lebepe [1,2] and Oluwatobi Samuel Oluwafemi [1,2,*]

[1] Department of Chemical Science, University of Johannesburg, Johannesburg 2028, South Africa
[2] Centre for Nanomaterials Sciences Research, University of Johannesburg, Johannesburg 2028, South Africa
* Correspondence: oluwafemi.oluwatobi@gmail.com

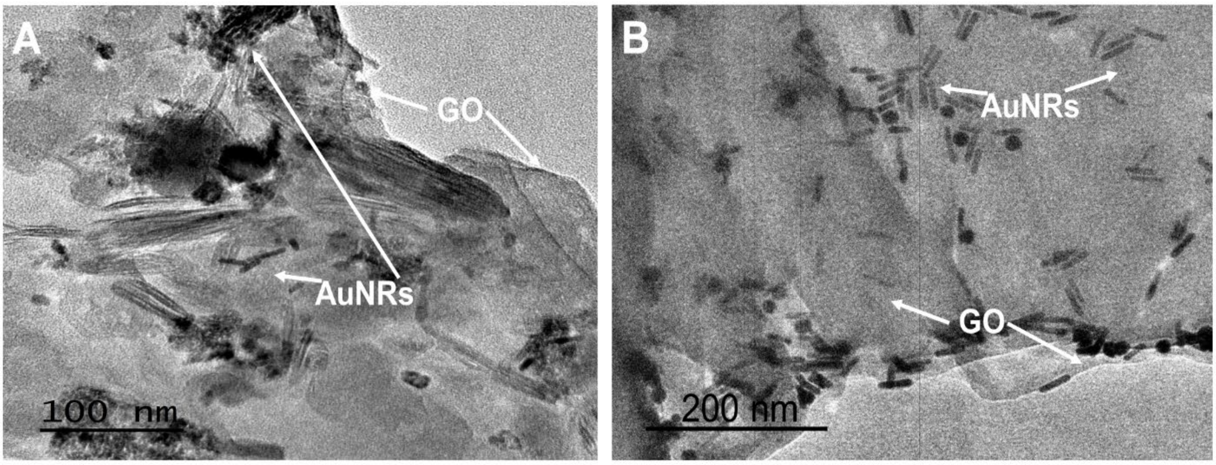

Figure S1. TEM images of GO@AuNRs and mGO@AuNRs

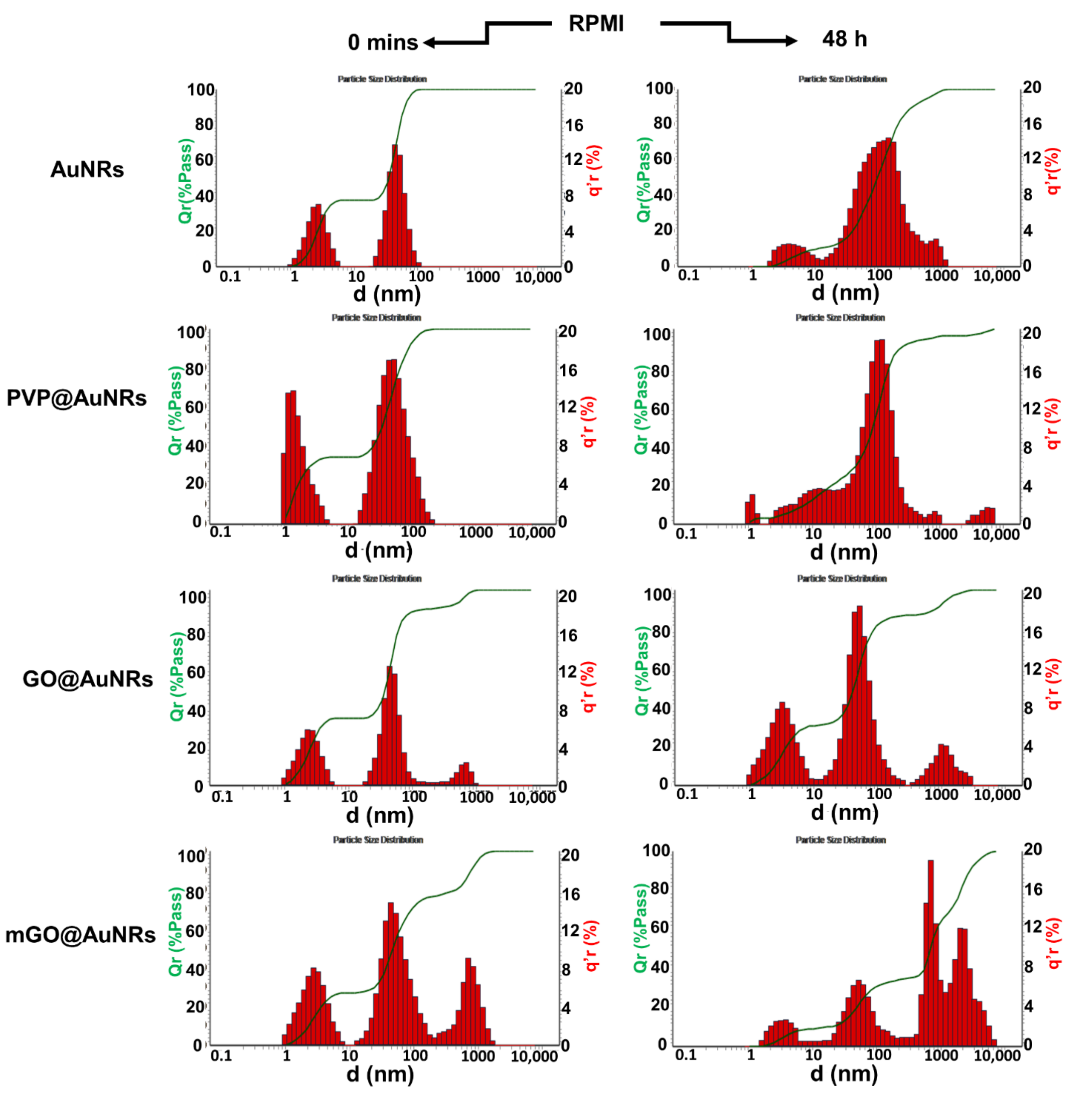

Figure S2. DLS of AuNRs, PVP @AuNRs, GO@AuNRs and mGO@AuNRs, in PBS at 0 min and 48 h.

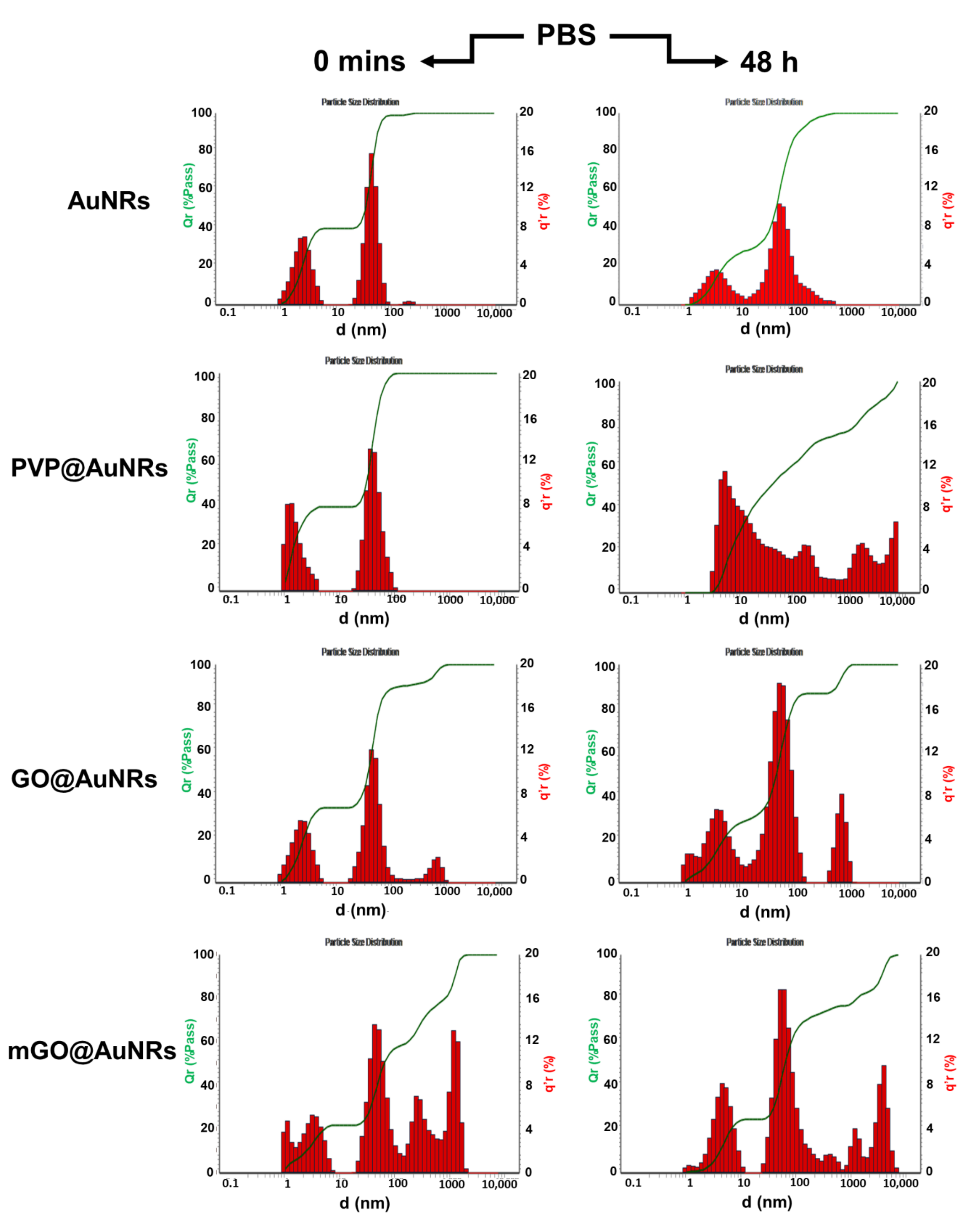

Figure S3. DLS of AuNRs, PVP @AuNRs, GO@AuNRs and mGO@AuNRs, in PBS at 0 min and 48 h.

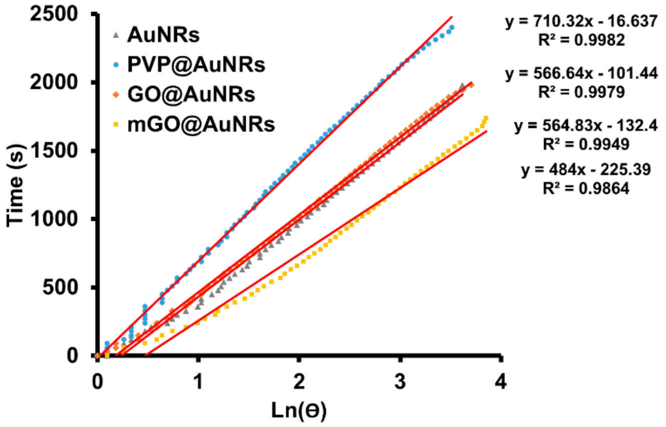

Figure S4. The time constant for heat transfer of AuNRs, PVP @AuNRs, GO@AuNRs, and mGO@AuNRs by applying the natural logarithm of temperature change versus time data acquired from the cooling.

Table S.1. Power density and phothermal conversion efficiency (%) of different nanocomposites irradiated with 808 nm laser.

Nanocomposites	Laser wavelength (nm) and power density	Photothermal conversion efficiency (%)	Ref
**Apt@GO@Au-His@a-ZnO**	1.5 W.cm^{-2}	38.0	[1]
**mPEG-PEI-AuNRs**	0.8 W cm^{-2}	27.7	[2]
**$MoTe_2$ nanosheets**	0.6 W cm^{-2}	33.8	[3]
**AuNRs/GO@PDA hybrid nanosheets**	2 W cm^{-2}	36.1	[4]
**PEG-AuNRs**	1.5 W cm^{-2}	52	[5]
**This work**<br>**PVP@AuNRs**<br>**GO@AuNRs**<br>**mGO@AuNRs**	1.27 W.cm^{-2}	 21.5 %<br>37.8 %<br>54.8%	-

References

1. Zhang, M.; Wu, F.; Wang, W.; Shen, J.; Zhou, N.; Wu, C. Multifunctional Nanocomposites for Targeted, Photothermal, and Chemotherapy. *Chem. Mater.* **2019**, *31*, 1847-1859. https://doi.org/10.1021/acs.chemmater.8b00934.
2. Xu, C.; Wang, Y.; Wang, E.; Yan, N.; Sheng, S.; Chen, J.; Lin, L.; Guo, Z.; Tian, H.; Chen, X. Effective Eradication of Tumors by Enhancing Photoacoustic-Imaging-Guided Combined Photothermal Therapy and Ultrasonic Therapy. *Adv. Funct. Mater.* **2021**, *31*, 2009314. https://doi.org/10.1002/adfm.202009314.
3. Ma, N.; Zhang, M.-K.; Wang, X.-S.; Zhang, L.; Feng, J.; Zhang, X.-Z. NIR Light-Triggered Degradable MoTe2 Nanosheets for Combined Photothermal and Chemotherapy of Cancer. *Adv. Funct. Mater.* **2018**, *28*, 1801139. https://doi.org/10.1002/adfm.201801139.
4. Qi, Z.; Shi, J.; Zhu, B.; Li, J.; Cao, S. Gold nanorods/graphene oxide nanosheets immobilized by polydopamine for efficient remotely triggered drug delivery. *Journal of Materials Science* **2020**, *55*, 14530-14543. https://doi.org/10.1007/s10853-020-05050-2.
5. Sun, M.; Liu, F.; Zhu, Y.; Wang, W.; Hu, J.; Liu, J.; Dai, Z.; Wang, K.; Wei, Y.; Bai, J. Salt-induced aggregation of gold nanoparticles for photoacoustic imaging and photothermal therapy of cancer. *Nanoscale* **2016**, *8*, 4452-4457.
